# Antimicrobial Susceptibility Profiles of *Escherichia coli* Isolates from Clinical Cases of Turkeys in Hungary (2022–2023)

**DOI:** 10.3390/antibiotics14040338

**Published:** 2025-03-25

**Authors:** Ákos Jerzsele, Ábel Szabó, Franciska Barnácz, Bence Csirmaz, László Kovács, Ádám Kerek

**Affiliations:** 1 Department of Pharmacology and Toxicology, University of Veterinary Medicine, István utca 2, 1078 Budapest, Hungary; jerzsele.akos@univet.hu (Á.J.); szabo.abel@student.univet.hu (Á.S.); barnacz.franciska@student.univet.hu (F.B.); csirmaz.bence@student.univet.hu (B.C.); 2 National Laboratory of Infectious Animal Diseases, Antimicrobial Resistance, Veterinary Public Health and Food Chain Safety, University of Veterinary Medicine, István utca 2, 1078 Budapest, Hungary; kovacs.laszlo@univet.hu; 3 Department of Animal Hygiene, Herd Health and Mobile Clinic, University of Veterinary Medicine, István utca 2, 1078 Budapest, Hungary; 4 Poultry-Care Kft., 5052 Újszász, Hungary

**Keywords:** *Escherichia coli*, antimicrobial resistance, minimum inhibitory concentration, MIC, poultry, turkeys, Hungary

## Abstract

Background: The global spread of antimicrobial resistance is one of the defining challenges of our time. Preserving the efficacy of antibiotics is a shared responsibility, which includes conducting regular surveillance studies. The poultry industry, which produces the highest quantity of animal-derived protein in the shortest time, faces significant challenges from *Escherichia coli*, a bacterium frequently responsible for clinical disease. Methods: This study aimed to assess the susceptibility of *E. coli* isolates collected from clinical cases in turkeys across Hungary to antibiotics of veterinary and public health importance using minimum inhibitory concentration (MIC) determination. Results: Over the course of one year, we analyzed a total of 70 clinical isolates. Most isolates (64.3%) were resistant to amoxicillin, and the observed 25.7% resistance to amoxicillin–clavulanic acid suggests that the majority of strains are beta-lactamase producers. The highest resistance level was observed against neomycin (74.3%). Resistance to critically important antibiotics, including enrofloxacin (28.6%), ceftriaxone (8.6%), and colistin (7.1%) raises significant public health concerns. When comparing our results with human hospital resistance data from Hungary, most findings showed comparable values, with the exception of neomycin, which exhibited markedly higher resistance in the isolates from poultry. Conclusions: Our findings underscore the necessity of regular surveillance studies, which should be periodically repeated in the future to observe temporal trends. These results should also be linked to antibiotic usage patterns, and the genetic background of multidrug-resistant strains should be further examined using next-generation sequencing techniques. This study provides critical insights into the current antimicrobial resistance landscape in the Hungarian poultry industry and highlights the urgent need for targeted interventions to prevent the dissemination of resistant strains to humans. The findings contribute valuable data for developing future AMR management strategies in veterinary and public health contexts.

## 1. Introduction

The spread of antimicrobial resistance (AMR) affects the entire planet. It is comparable to climate change in that the effects of AMR in one region may be felt in another, impacting both animal and public health. Also akin to climate change, the declining efficacy of antibiotics poses a particular challenge in low- and middle-income countries, which is likely to be a defining issue in the coming decades [1].

*Escherichia coli* is one of the most significant pathogens impacting poultry production worldwide. The avian pathogenic *E. coli* (APEC) O78 strain is responsible for annual global economic losses amounting to millions of dollars due to high mortality rates and the associated reduction in production efficiency [2,3,4]. *E. coli* O157:H7 is a foodborne pathogen that predominantly causes diarrhea, with potentially life-threatening outcomes due to Shiga toxin production [5,6,7]. In poultry meat production, the presence of *E. coli* is typically an indicator of microbiological quality, fecal contamination, or inadequate cleaning and general hygiene practices [8,9]. It is a Gram-negative bacterium that naturally resides as a commensal in the gastrointestinal tracts of humans and animals, contributing to the formation of the antimicrobial resistome in the gut microbiome.

The commensal microbiome’s diversity plays a significant role in the development and dissemination of AMR [10,11,12]. Resistant genes present within these bacteria can be horizontally transferred to other pathogens [13,14], potentially complicating treatment options and increasing the risk of treatment failure [15]. Although the majority of *E. coli* strains are non-pathogenic, some can cause various clinical diseases in poultry, such as colisepticaemia, airsacculitis, cellulitis, peritonitis, and synovitis [13,16,17,18].

Thus, proper biosecurity measures on livestock farms play a critical role [19]. Following the swine sector [20], the poultry industry is the second-largest consumer of antibiotics [21], making responsible and compliant antibiotic use within this sector particularly important. To preserve the efficacy of these active compounds, susceptibility testing should be conducted prior to therapeutic treatments. Combined with appropriate pharmacokinetic and pharmacodynamic analyses, these tests allow for the selection of the most effective therapy [22]. A 2012 study reported a high prevalence of AMR among *E. coli* isolates from conventionally raised turkey meat, which suggests that antimicrobial use may still be higher in traditional turkey production systems compared to modern production methods [23].

In recent years, research has increasingly focused on finding alternative solutions to combat AMR. Promising alternatives include plant essential oils, antimicrobial peptides, and propolis, each of which offers unique mechanisms of action. Plant essential oils and extracts have demonstrated antimicrobial properties through mechanisms such as disrupting cell membranes and inhibiting enzyme activity [24]. Antimicrobial peptides can effectively target bacterial cells by interacting with their membranes, causing structural damage and subsequent cell death [25]. Propolis, a resinous mixture produced by bees, exhibits broad-spectrum antimicrobial activity largely due to its high flavonoid content, which interferes with bacterial metabolism and cell wall synthesis [26,27,28]. Furthermore, medium-chain fatty acids and triglycerides have shown antimicrobial efficacy by adhering to bacterial cell walls and causing plasma leakage [29].

The increasing need to find alternatives to antibiotics highlights the urgency of addressing the AMR issue. While antibiotics remain essential for the treatment of bacterial infections, overuse and misuse are accelerating resistance development. Therefore, it is crucial to integrate alternative approaches into current treatment protocols to enhance their efficacy and reduce selective pressure on bacterial populations.

In accordance with the Commission Implementing Decision 2013/652/EU [30], AMR was monitored in 2019–2020 by examining *E. coli* strains isolated from cecal contents collected during the slaughter of key food-producing animals. Data were provided by twenty-seven EU member states and five non-member countries during this survey period. The monitoring of broiler chicken and turkey isolates occurred in 2020, during which the prevalence of certain genes was assessed, including extended-spectrum beta-lactamase (ESBL) and *ampC* genes, as well as carbapenemase-producing strains [30,31].

The findings showed that, among poultry in the EU, AMR was widespread. Multidrug-resistant (MDR) isolates accounted for 38.7% of broiler isolates and 41% of turkey isolates. Resistance to ciprofloxacin and nalidixic acid was common, and critical resistance levels were also reported for chloramphenicol and gentamicin. Resistance to fluoroquinolones was found to be alarmingly high (60.7% for ciprofloxacin and 51.8% for nalidixic acid in broilers; 33.5% and 22.9%, respectively, in turkeys). However, in contrast, colistin resistance was uncommon, and resistance to cefotaxime and ceftazidime was reported at 0.5% and 0.4% in chickens and 1.4% and 1.0% in turkeys, respectively [32]. The prevalence of ESBL was 1%, and *ampC* gene carriage was 0.3% in broilers, compared to 1% and 0.4% in turkeys [32].

Given the significant economic importance of the poultry industry, routine and comprehensive surveillance studies are vital. Therefore, we aimed to assess the antimicrobial resistance profiles of *E. coli* strains isolated from clinical cases in large-scale turkey populations in Hungary. These strains were tested against antibiotics relevant to both veterinary and public health. Regular surveys such as this contribute to the development of responsible antibiotic use practices.

The responsible use of antimicrobials is a key issue in the One Health approach, which emphasizes the interconnected health of animal plants and their combined impact on human health globally [33]. The One Health approach stresses the importance of evidence-based medicine; thus, efforts to collect data on AMR play a crucial role in the long-term management of this global issue [33,34].

## 2. Results

### 2.1. Regional Distribution and Origin of Samples Received

A total of 70 *E. coli* strains were isolated for antimicrobial resistance testing from clinical cases in large-scale turkey flocks. Of these isolates, 47.1% originated from the Dél-Alföld region, 2.9% from the Dél-Dunántúl region, 24.3% from the Észak-Alföld region, 4.3% from the Észak-Magyarország region, 8.6% from the Közép-Dunántúl region, 1.4% from the Közép-Magyarország region, and 11.4% from the Nyugat-Dunántúl region. Regarding the source organ, 51.4% of the strains were isolated from bone marrow, 27.2% from the liver, 18.6% from the lungs, 1.4% from the trachea, and 1.4% from eggs. These isolates were obtained by the national reference laboratory staff using Chromobio *E. coli* agar (Biolab Zrt., Budapest, Hungary).

### 2.2. Antimicrobial Susceptibility Testing

**Figure 1:**
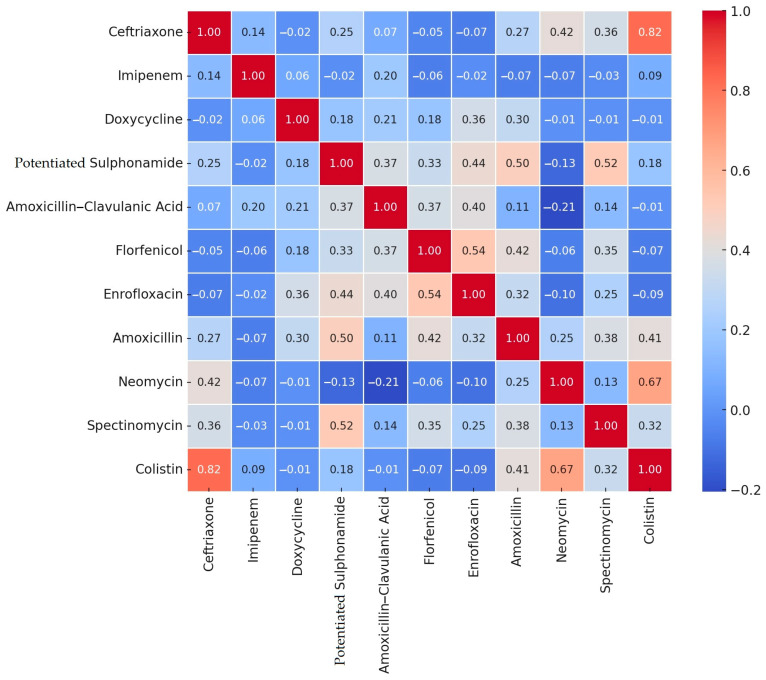
A correlation study between antibiotic agents based on the resistance profile of *Escherichia coli* strains of turkey origin isolated from clinical cases, plotted on a heat map.

We determined the minimum inhibitory concentration (MIC) values of the isolates for 15 antibiotics of veterinary and public health importance, of which 11 had clinical breakpoints. For these, we calculated the proportion of resistant strains and conducted correlation analyses among the antibiotics. The results were visualized as a heatmap (Figure 1).

The most significant positive correlation was observed between ceftriaxone and colistin (0.82), suggesting potential cross-resistance or co-selection mechanisms. Additionally, colistin and neomycin showed a strong positive correlation (0.67), indicating that these antibiotics may have related resistance mechanisms. Moderate positive correlations were detected between florfenicol and enrofloxacin (0.54) and between potentiated sulfonamides and spectinomycin (0.52), which could suggest similar resistance pathways. Negative correlations, such as between neomycin and amoxicillin–clavulanic acid (−0.21), highlight possible antagonistic effects between these antibiotics. Understanding these correlations is essential for establishing potential co-selection or cross-resistance mechanisms, which may impact antibiotic treatment efficacy.

Correlation analyses are crucial for identifying relationships between the factors studied, which can help define the direction of research or support a hypothesis. However, it is important to note that correlation does not imply causation; it merely indicates that a relationship exists between the variables.

**Figure 2:**
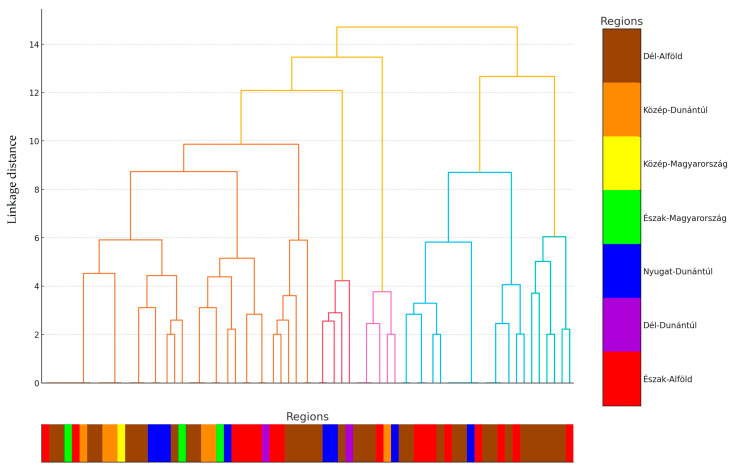
The results of the hierarchical cluster analysis on a dendrogram. Clustering was based on the antimicrobial resistance profile of each strain. To make the data points more meaningful, each sample was assigned to the corresponding region of origin, regions were color-coded, and the corresponding color code for the sample was indicated by lines below the horizontal axis.

Figure 2 illustrates the results of the hierarchical cluster analysis, visualized as a dendrogram. The analysis aimed to identify similarities and differences between the isolates based on their resistance profiles. To enhance interpretability, each sample was color-coded according to its region of origin. The majority of samples originated from the Dél-Alföld region, as indicated by the prevalence of brown-coded data points. The clustering tendency suggests that local environmental or management factors may influence antimicrobial resistance development. The dendrogram also highlights three major clusters of isolates, which are further investigated in the subsequent principal component analysis (PCA).

**Figure 3:**
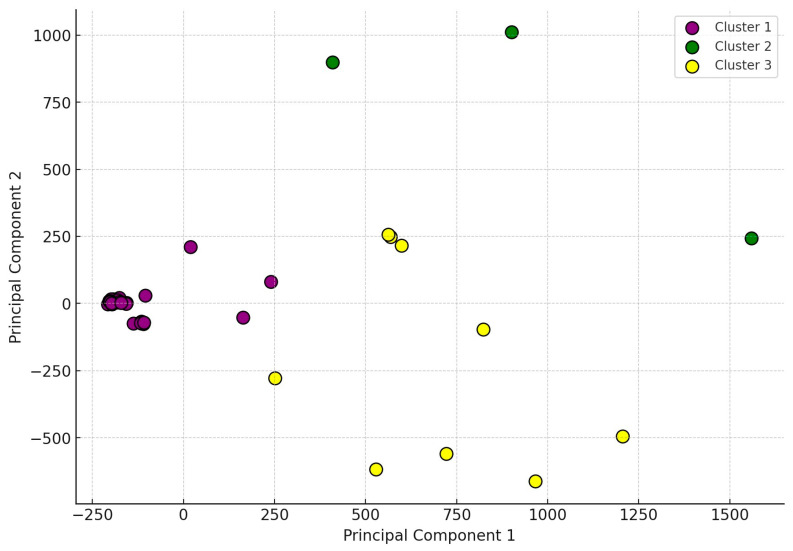
Principal component analysis of strains (*n* = 70) causing clinical disease in turkeys. The matrix analysis of variables was performed by examining their joint and differential variation and plotting them in a coordinate system.

The PCA plot (Figure 3) provides a visual representation of the relationships between the identified clusters. The clustering highlighted in purple (Cluster 1) is distinctly separated from the other two clusters, suggesting a unique resistance pattern that may be attributed to specific regional practices or genetic factors. The green (Cluster 2) and yellow (Cluster 3) clusters are more scattered, indicating variability in resistance profiles among the isolates. PCA is a crucial tool for reducing the dimensionality of complex datasets and providing insight into how various factors may contribute to observed patterns.

MIC values were determined for 15 antibiotics of veterinary and public health significance, 11 of which had clinical breakpoints. The frequencies of MIC values, along with calculated MIC_50_ and MIC_90_ values and the European Committee on Antimicrobial Susceptibility Testing (EUCAST) defined epidemiological cut-off (ECOFF) values, are summarized in Table 1. The frequency table for active substances without a clonal breakpoint is summarized in Supplementary Table S1.

The detailed presentation of previous figures, as well as Table 1, is essential to provide a comprehensive understanding of the antimicrobial resistance landscape. The correlation analysis (Figure 1) identifies potential relationships between antibiotics, which is crucial for guiding further research and improving therapeutic strategies. The hierarchical cluster analysis (Figure 2) and PCA (Figure 3) provide complementary insights by highlighting regional differences and clustering patterns, which may be driven by distinct environmental or management factors. Finally, Table 1 provides an in-depth overview of MIC distributions, enabling a better understanding of resistance mechanisms and the extent of antimicrobial resistance among the isolates. Together, these results provide a solid foundation for discussing the implications of antimicrobial resistance in poultry farming and developing targeted interventions.

When examining MIC_50_ and MIC_90_ values, they were below the clinical breakpoints for ceftriaxone, imipenem, and colistin. Apart from imipenem and colistin, the MIC_50_ values were also below the clinical breakpoints for amoxicillin–clavulanic acid, spectinomycin, doxycycline, and enrofloxacin. MIC_50_ represents the minimum antibiotic concentration required to inhibit the growth of 50% of the tested bacterial strains, while MIC_90_ represents the concentration needed to inhibit 90%. These values provide insight into the effectiveness of an antibiotic against a bacterial species on a population level. The lower these values are, the more effective the antibiotic is.

When compared to the ECOFF values, both MIC_50_ and MIC_90_ values were below the for colistin. MIC_50_ values were also below for ceftriaxone and imipenem. ECOFF is an epidemiological cut-off value used in antimicrobial resistance studies to distinguish naturally susceptible bacterial strains from those that potentially carry resistance mutations or genes. ECOFF serves as a threshold that separates “wild type” bacterial strains (those without any acquired resistance) from strains with mutations or resistance mechanisms that contribute to antimicrobial resistance.

The significance of ECOFF values in veterinary practice lies in their ability to inform antibiotic selection by identifying potential resistance even before clinical resistance emerges. Since clinical breakpoints are primarily established based on therapeutic efficacy in humans, ECOFF values provide critical insights specific to veterinary contexts. For instance, if MIC values are consistently above the ECOFF but below clinical breakpoints (ceftriaxone and imipenem), it may suggest the early emergence of resistance, which could compromise treatment efficacy over time. Therefore, monitoring ECOFF values can guide veterinary practitioners in selecting antibiotics that maintain efficacy and prevent the development of resistant strains. Additionally, it helps establish more accurate guidelines for antibiotic use in animals, especially in food-producing species, thereby supporting both animal health and public health objectives.

**Figure 4:**
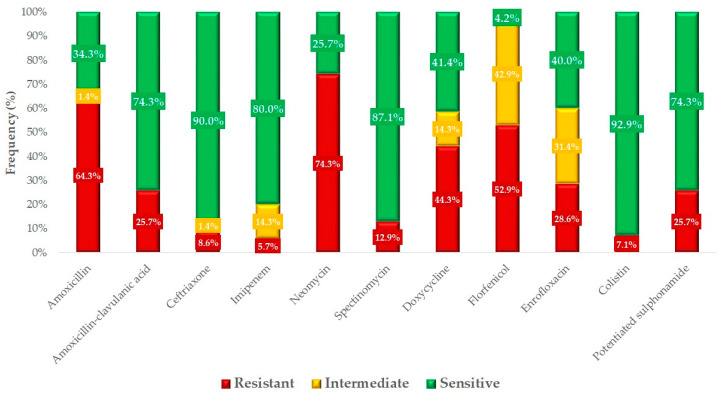
Antimicrobial resistance profile of turkey *Escherichia coli* samples (*n* = 70) collected from clinical cases.

Based on the clinical breakpoints, we determined the percentage of isolates classified as resistant, intermediate, or susceptible (Figure 4). Overall, 64.3% of the isolates were resistant to amoxicillin, while 25.7% were resistant to amoxicillin–clavulanic acid, indicating that a significant proportion of the strains produce beta-lactamase. The highest resistance was observed against neomycin (74.3%), followed by florfenicol (52.9%) after amoxicillin. The resistance levels observed for critically important antibiotics were concerning ceftriaxone (8.6%), colistin (7.1%), and enrofloxacin (28.6%).

**Figure 5:**
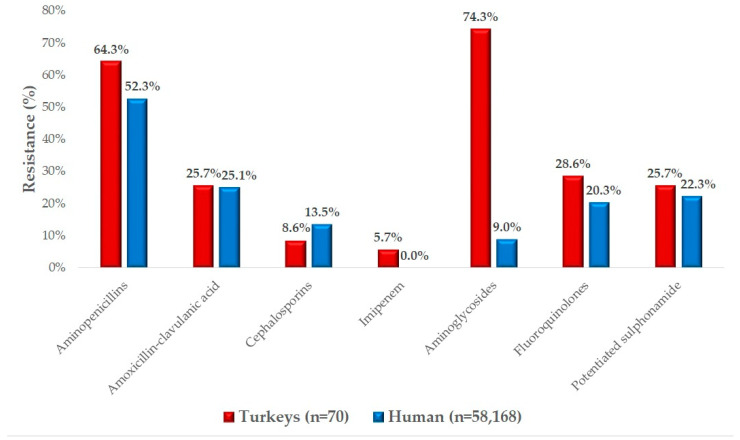
Comparison of resistance profiles of *Escherichia coli* strains causing clinical disease isolated from turkeys and human resistance data.

We had the opportunity to compare our results with human resistance data (Figure 5). Resistance rates for amoxicillin in veterinary medicine and ampicillin in human healthcare were found to be very similar. Comparable values were also observed for amoxicillin–clavulanic acid. For cephalosporins, resistance was lower in turkey isolates (8.6%) compared to human hospital data (13.5%). However, the 5.7% resistance observed against imipenem in turkey isolates is concerning, as no resistant strains were detected in the human data. Resistance to aminoglycosides was remarkably high in veterinary samples (74.3%) compared to the 9.0% resistance observed in human samples. Resistance rates for fluoroquinolones and potentiated sulfonamides were similar across both animal and human samples.

## 3. Discussion

This study provides a comprehensive overview of the antimicrobial resistance profiles of *E. coli* isolates from turkeys in Hungary. A nationwide susceptibility study was conducted on *E. coli* strains isolated from clinical cases in Hungary (*n* = 70) using MIC determination. Our findings indicate a high prevalence of resistance against several antibiotics commonly used in veterinary practice, particularly aminopenicillins, aminoglycosides, florfenicol, and tetracyclines.

The current global public health threat of AMR [34,35] presents an increased risk of resistance during hospital treatments [36]. Resistance is becoming increasingly critical not only in *E. coli* strains originating from turkeys but also in *Campylobacter* and *Salmonella* species [37,38,39]. Data related to chickens cannot be extrapolated to the turkey sector due to the differing operational practices of the industry [26], which further supports the justification for a separate assessment of the AMR situation in turkeys. This research highlights important correlations regarding resistance to specific antibiotics, as well as the population-level resistance status. The findings presented are of significant importance to the sector as they address existing knowledge gaps.

The results revealed that 64.3% of the strains were resistant to amoxicillin. Shrestha et al. (2022) reported a resistance rate of 31.2% [40], while Suwono et al. (2021) documented 67.6% resistance to ampicillin [41]. Agunos et al. observed an increase in resistance from 50% to 70% over a four-year period [42]. Our findings align with other international studies. The high resistance rate is likely attributed to the decades-long and widespread use of amoxicillin in the poultry industry.

The 25.7% resistance to amoxicillin–clavulanic acid, which we observed, suggests frequent β-lactamase production in the Enterobacteriaceae family. Boulianne et al. (2016) reported an even higher resistance rate of 34.5% [43]. Although clavulanic acid does not have an established maximum residue limit (MRL) and is therefore not permitted for use in the poultry sector, monitoring resistance to this compound in in vitro studies is particularly important from a public health perspective.

Cephalosporins are not used in turkeys; however, the continuous increase in resistance to these compounds poses a significant problem in human medicine. We observed an 8.6% resistance rate to ceftriaxone, whereas Cook et al. (2009) found no resistant strains [44]. In contrast, Boulianne et al. (2016) reported a significantly higher resistance rate of 33.1% [43], and Agunos et al. (2019) documented a 1–10% increase over a comprehensive four-year study [42]. The presence of resistance to cephalosporins may be associated with the previously permitted use of third-generation ceftiofur, administered in hatcheries via egg or day-old poultry injections. Another contributing factor could be the development of cross-resistance with other antimicrobial agents.

We observed a 44.3% resistance rate to doxycycline, while Shrestha et al. (2022) reported a higher resistance rate of 61.7% [40], and Grobbel et al. (2022) documented a resistance rate of 49% [45]. The extensive use of tetracyclines over several decades, along with their poor oral bioavailability, likely contributed to the high resistance rates.

The rate of resistance for neomycin was 74.3%, while Shrestha et al. (2022) reported a 45% resistance rate to aminoglycosides [40], and Boulianne et al. (2016) documented a 27.9% resistance rate to neomycin [43], In contrast, Grobbel et al. (2022) observed only a 5% resistance rate to gentamicin [45], and Suwono et al. (2021) reported an 11.8% resistance rate [41]. Agunos et al. (2019), during a four-year study, noted an increase in resistance rates from 10% to 30% [42]. The extensive use of aminoglycosides over decades is a potential contributor to the high resistance observed against neomycin. It is also important to highlight its practical implications, as cross-resistance within the aminoglycoside class has been well-documented [46].

In our study, the resistance rate to enrofloxacin was 28.6%, which is a critically important antimicrobial agent. The use of fluoroquinolones, including enrofloxacin, must be significantly reduced in the future, particularly as the poultry industry is one of the largest consumers of this class of antibiotics. Interestingly, Boulianne et al. in 2016 [43] and Cook et al. in 2009 [44] did not detect resistance to fluoroquinolones, whereas Grobbel et al. (2022) reported a 35% resistance rate to ciprofloxacin [45], and Suwono et al. (2021) noted a 28% resistance rate [41]. The use of enrofloxacin carries substantial public health implications, as a proportion of it metabolizes into ciprofloxacin in the body—a compound primarily utilized in human healthcare.

We observed a 25.7% resistance rate to potentiated sulfonamides (trimethoprim and sulfamethoxazole in a 1:19 ratio). In comparison, Shrestha et al. (2022) reported a 30.4% resistance rate [40], while Boulianne et al. (2016) documented a significantly higher resistance rate of 82.7% [43]. This antimicrobial class has a long history of use spanning several decades. However, its utilization has declined in recent years, allowing the resurgence of wild-type strains, which are gradually becoming more widespread [47].

We identified a 7.1% resistance rate to colistin. Nobili et al. (2022) reported a 3.2% resistance rate in slaughterhouse samples [48], while Mesa-Varona et al. (2020) initially did not identify any resistant strains; however, four years later, they documented a resistance rate of 9.5% [49]. The slow but steady increase in colistin resistance is particularly concerning, as this critically important antimicrobial must be preserved for future generations to ensure its effectiveness.

We observed a resistance rate of 52.9% to florfenicol. Hu et al. (2022) reported 100% resistance in environmental strains [50], while Gambi et al. (2022) did not identify any resistant strains [51]. These findings further underscore the critical role of commensal strains, alongside clinical strains, as reservoirs that develop and maintain resistance. For spectinomycin, we identified a resistance rate of 12.9%. In 2014, Piccirillo et al., analyzing turkey meat, reported a resistance rate of 4.7% [52]. The 5.7% resistance rate observed for imipenem is concerning; Moawad et al. (2022) did not detect any resistant strains [53]. The 5.7% resistance rate to imipenem observed in veterinary isolates, despite its absence in human data, likely reflects cross-resistance arising from the use of other antibiotics, as imipenem itself is not used in veterinary medicine. However, the instability of imipenem in aqueous environments highlights the need for future investigations using more stable compounds.

The significant correlation observed between certain antibiotics, such as ceftriaxone and colistin, and neomycin and colistin, suggests potential cross-resistance or co-selection mechanisms that warrant further investigation.

Variations in rearing duration and antibiotic usage could lead to substantial differences in antimicrobial resistance. This is supported by our previous study on commensal strains [54]. In the future, it may be worthwhile examining clinical strains based on production purposes to determine if there are statistically significant differences in resistance levels.

The European Food Safety Authority (EFSA) monitoring programs [55,56,57,58,59] for European Union member states reported a decrease in aminopenicillin resistance in turkeys from 69.0% in 2014 to 55.8% in 2022. The 64.3% resistance rate observed in our study can be considered an intermediate value within the period examined. Regarding cephalosporins, a similar decreasing trend was reported by EFSA, from 2.3% to 1.4%, while our findings indicate a substantially higher resistance rate of 8.6%. This discrepancy may suggest regional differences or specific selection pressures present in the Hungarian poultry sector.

In the case of fluoroquinolones, EFSA reported a decrease from 50.3% to 40.0%, which remains significantly higher than the 28.6% resistance rate we detected. This observation suggests that the use of fluoroquinolones may be more restricted or regulated in our region compared to other areas of the European Union. Tetracyclines also showed a significant decline in resistance from 70.9% to 52.0% during the same period, with our study reporting an even lower resistance rate of 44.3%.

Conversely, the continuous decline in colistin resistance from 7.4% to 3.6%, as reported by EFSA, is not consistent with our findings of 7.1%. This inconsistency may indicate local differences in the use of colistin or the presence of resistant clones circulating within the Hungarian turkey population.

Comparing our results with those reported from neighboring Romania, resistance rates for *E. coli* isolates from chicken meat were significantly higher. Ampicillin resistance was found to be 80.0%, ciprofloxacin 56.7%, cefotaxime 46.7%, and gentamicin 40.0% [60]; these values, with the exception of neomycin, are considerably higher than those detected in our study. This disparity further emphasizes the necessity of continuous surveillance and the importance of regional comparisons to understand the broader epidemiological landscape of antimicrobial resistance in poultry.

When comparing the Hungarian human resistance data with our findings, we observed that the resistance rates for amoxicillin, used in veterinary medicine, and ampicillin, monitored in human medicine, were similar but significantly higher than those for amoxicillin–clavulanic acid in both cases. This indicates that a significant proportion of the strains produce β-lactamase. Carmona-Cartaya et al. (2022) reported 68.3% resistance to ampicillin in hospital strains [61], which is comparable to the 64.3% we observed in turkey strains and the 52.3% resistance noted in public health data.

The significantly higher resistance observed for aminoglycosides in both human and animal samples reflects their decades-long widespread use in both human medicine and veterinary practice. However, the remarkably high resistance (74.3%) observed in turkey isolates compared to the 9.0% resistance in human samples suggests that veterinary use of aminoglycosides, particularly neomycin, remains extensive and problematic.

While resistance levels for fluoroquinolones and potentiated sulfonamides were also comparable between sectors, the similarities between these two domains highlight potential transmission pathways. The close contact between humans and poultry, especially within the food production chain, increases the risk of resistant strains spreading from animals to humans. This finding emphasizes the importance of monitoring antimicrobial resistance in animals as a crucial part of a One Health approach.

Additionally, the observation of 5.7% resistance to imipenem in turkey isolates, with no corresponding resistance detected in human samples, is particularly alarming. This finding suggests that the selection pressure exerted by the veterinary use of antimicrobials may contribute to the development of resistant strains that have not yet emerged in human clinical settings.

The comparison between human and animal resistance data serves as an essential tool to identify overlapping resistance profiles and possible cross-contamination or transmission routes. Understanding the relationship between these findings is critical for developing targeted interventions aimed at reducing the spread of antimicrobial resistance between animals and humans. Future studies should explore these relationships further to establish specific measures to mitigate the dissemination of resistant strains.

These findings are significant because, in human healthcare, amoxicillin is often a first-choice treatment for urinary tract infections caused by *E. coli* [62], while fluoroquinolones and potentiated sulfonamides are the primary options for treating prostatitis [63,64]. Without them, treatment options become more limited, and this could further exacerbate the development of resistance in *E. coli* strains. Preserving the efficacy of these antibiotics is, therefore, critical for future generations.

Future studies should focus on characterizing the underlying resistance mechanisms, particularly those related to colistin and imipenem, as well as evaluating the role of management practices and antibiotic usage patterns in shaping resistance profiles. Moreover, the distinction between commensal and clinical strains should be further explored to determine their respective contributions to the overall resistance burden.

The findings presented in this study contribute to a better understanding of the antimicrobial resistance landscape in poultry farming and provide valuable insights for developing effective mitigation strategies. Given the critical importance of certain antibiotics for human health, it is essential to implement targeted interventions aimed at reducing antimicrobial use and minimizing the spread of resistant strains within both animal populations and the broader environment.

## 4. Materials and Methods

### 4.1. The Origin of Strains and Human Data

The examined strains were submitted between February 2022 and May 2023 to the National Food Chain Safety Office’s Veterinary Diagnostic Directorate by veterinarians serving poultry farms. The samples were collected from deceased animals that succumbed to illnesses. Subsequently, the staff at the reference laboratory identified the strains using Chromobio agar (Biolab Zrt., Budapest, Hungary) based on the formation of purple colonies. The resulting pure cultures were provided for further analysis and were cryopreserved at −80 °C using the Microbank™ system (Pro-Lab Diagnostics, Richmond Hill, ON, Canada).

The Hungarian human antimicrobial resistance data from 2022 were made available by the National Public Health and Pharmaceutical Center with the authorization of the National Chief Medical Officer.

### 4.2. Minimum Inhibitory Concentration (MIC) Determination

Phenotypic expression of AMR was determined by assessing the MIC values for each bacterial strain according to the methodology of the Clinical Laboratory Standard Institute (CLSI) [65]. The breakpoints were also defined based on CLSI guidelines [66] and compared with the ECOFF values established by the EUCAST.

The bacterial strains stored at −80 °C were suspended in 3 mL of cation-adjusted Müller–Hinton broth (CAMHB) (Biolab Zr., Budapest, Hungary) one day prior to testing and incubated at 37 °C for 18–24 h. The MIC determination was conducted using 96-well microtiter plates (VWR International, LLC., Debrecen, Hungary). Except for the first column, all wells of the working plates were filled with 90 µL of CAMHB. The stock solutions of the tested antimicrobial agents (Merck KGaA, Darmstadt, Germany) were prepared at 1024 µg/mL according to CLSI guidelines [66].

A bacterial suspension adjusted to a 0.5 McFarland standard using a nephelometer (ThermoFisher Scientific, Budapest, Hungary) was inoculated into the microtiter plates, starting from the 11th column backward, with 10 µL per well [65]. The evaluation was performed using a Sensititre™ SWIN™ automatic MIC reader (ThermoFisher Scientific, Budapest, Hungary) and VIZION system software version 3.4 (ThermoFisher Scientific, Budapest, Hungary, 2024). The reference isolate used was *Escherichia coli* (ATCC 25922).

### 4.3. Statistical Analysis

The purpose of correlation analysis is to explore the degree and direction of the relationship between two or more variables. This statistical method reveals how variables move together: whether an increase in one variable corresponds to an increase or decrease in the other. The correlation strength is measured on a scale from −1 to +1, where +1 indicates a strong positive relationship, −1 is a strong negative relationship, and 0 signifies no linear relationship between the variables [67].

Hierarchical cluster analysis [68,69] is a method used to group data points into clusters based on their similarities. This approach is particularly useful when the number of clusters is unknown beforehand, as it builds the clusters step by step. Initially, each data point is treated as its own cluster. The method then interactively combines the closest (most similar) data points or clusters until the desired number of clusters is achieved or a specified distance threshold is reached. The data points are represented in a multidimensional space, with the distances (similarities or differences) between them measured using a metric. The results are visualized using a dendrogram, a tree-like diagram that illustrates how the clusters are formed and connected. By cutting the dendrogram to a certain level, the number of clusters can be determined, with each branch representing a distinct cluster.

PCA is a dimensionality reduction technique used to simplify large, multidimensional datasets into a more manageable form while retaining as much information as possible from the original data [70]. This method is particularly useful when correlations among many variables lead to redundancy, allowing these dimensions to be reduced to a few principal components. To perform PCA, the covariance matrix of the data matrix is calculated. This square matrix shows how the variables change together. From the covariance matrix, eigenvectors and eigenvalues are derived. Eigenvectors represent the directions with the most variance in the data (these are the principal components), while eigenvalues indicate how much variance is explained by each principal component. The first few principal components account for the largest portion of the data’s variance. These components are selected for dimensionality reduction, reducing the number of dimensions to far fewer than the original number of variables while retaining most of the information. This enables the data to be visualized and interpreted more effectively.

## 5. Conclusions

The continuous monitoring of antimicrobial resistance, establishing temporal trends, and identifying patterns are essential in combatting the increasing spread of antimicrobial resistance. While general surveillance provides valuable data, this study highlights the necessity of focusing specifically on resistance mechanisms related to critically important antibiotic classes, such as cephalosporins, fluoroquinolones, and polymyxins.

Future research should include larger sample sizes to enhance statistical reliability and improve the identification of resistance trends. Additionally, it is essential to investigate resistance to a broader range of antimicrobial agents and incorporate detailed data on antibiotic usage, particularly focusing on the potential cross-resistance mechanisms associated with carbapenems and colistin. Employing next-generation sequencing for multidrug-resistant strains would also provide crucial insights into the genetic determinants of resistance and help distinguish between acquired and intrinsic resistance mechanisms.

Collectively, these findings will support the implementation of the One Health approach, promoting a comprehensive strategy to address antimicrobial resistance across interconnected domains of human, animal, and environmental health.

## Figures and Tables

**Table 1:** Frequency table of minimum inhibitory concentration (MIC) values for active substances with breakpoints in *Escherichia coli* samples of turkey origin (*n* = 70). The top row of each agent shows the number of pieces, and the bottom row shows each percentage. The red vertical line indicates the clinical breakpoint.

Antibiotic	^1^ BP *	0.001	0.002	0.004	0.008	0.016	0.03	0.06	0.125	0.25	0.5	1	2	4	8	16	32	64	128	256	512	1024	MIC_50_	MIC_90_	^2^ ECOFF
	µg/mL																						µg/mL		
Enrofloxacin	^1^ 2					4	16	1	2	5	13	9	1	3	3	11	1	1					0.5	16	0.125
						5.7%	22.9%	1.4%	2.9%	7.1%	18.6%	12.9%	1.4%	4.3%	4.3%	15.7%	1.4%	1.4%							
Colistin	2		1	0	0	5	3	5	8	9	19	15	5										0.5	1	2
			1.4%	0.0%	0.0%	7.1%	4.3%	7.1%	11.4%	12.9%	27.1%	21.4%	7.1%												
Ceftriaxone	^1^ 4					5	19	21	8	3	5	2	1	0	0	4	0	0	0	0	2		0.06	1	0.125
						7.1%	27.1%	30.0%	11.4%	4.3%	7.1%	2.9%	1.4%	0.0%	0.0%	5.7%	0.0%	0.0%	0.0%	0.0%	2.9%				
Imipenem	^1^ 4						1	4	10	26	11	4	10	4									0.25	2	0.5
							1.4%	5.7%	14.3%	37.1%	15.7%	5.7%	14.3%	5.7%											
^3^ Potentiated sulphonamide	^1^ 4										2	3	6	10	14	15	2	0	11	0	2	5	8	128	0.5
											2.9%	4.3%	8.6%	14.3%	20.0%	21.4%	2.9%	0.0%	15.7%	0.0%	2.9%	7.1%			
Doxycycline	^1^ 16										5	4	11	9	10	19	9	2	1				8	32	8
											7.1%	5.7%	15.7%	12.9%	14.3%	27.1%	12.9%	2.9%	1.4%						
Florfenicol	^1^ 16													3	30	17	5	11	0	2	2		16	64	16
														4.3%	42.9%	24.3%	7.1%	15.7%	0.0%	2.9%	2.9%				
Amoxicillin	^1^ 32										1	0	4	14	5	1	29	0	1	5	3	7	32	512	8
											1.4%	0.0%	5.7%	20.0%	7.1%	1.4%	41.4%	0.0%	1.4%	7.1%	4.3%	10.0%			
^4^ Amoxicillin–clavulanic acid	32										6	1	2	9	13	21	17	1					16	32	8
											8.6%	1.4%	2.9%	12.9%	18.6%	30.0%	24.3%	1.4%							
Neomycin	32											1	2	2	1	12	39	10	0	0	3		32	64	8
												1.4%	2.9%	2.9%	1.4%	17.1%	55.7%	14.3%	0.0%	0.0%	4.3%				
Spectinomycin	128														1	4	44	12	4	2	2	1	32	128	64
															1.4%	5.7%	62.9%	17.1%	5.7%	2.9%	2.9%	1.4%			

* BP—breakpoint; ^1^ Clinical Laboratory Standard Institute (CLSI); ^2^ Epidemiological cut-off value (EUCAST); ^3^ trimethoprim—sulfamethoxazole 1:19 ratio; ^4^ 2:1 ratio.

## Data Availability

The data presented in this study are available from the corresponding author upon reasonable request.

## Supplementary Materials

The following supporting information can be downloaded at https://www.mdpi.com/article/10.3390/antibiotics14040338/s1. Table S1: Frequency table of the minimum inhibitory concentration (MIC) values (µg/mL) for agents without breakpoints in *Escherichia coli* samples derived from turkeys (*n* = 70).
