# Supplementary material for: Antimicrobial Susceptibility Profiles of Escherichia coli Isolates from Clinical Cases of Turkeys in Hungary (2022–2023)
Source: Antibiotics (Basel). 2025 Mar 25;14(4):338. doi: 10.3390/antibiotics14040338 (PMC12024004; doi:10.3390/antibiotics14040338)
Supplement: Supplementary file 1 [file antibiotics-14-00338-s001.zip › antibiotics-3545048-supplementary.pdf]

Supplementary Table S1 Frequency table of the minimum inhibitory concentration (MIC) values (µg/mL) for agents without breakpoints in *Escherichia coli* samples derived from turkeys ($n = 70$). The top row for each agent shows the count, while the bottom row shows the percentage.

Antibiotic	0.001	0.002	0.004	0.008	0.016	0.03	0.06	0.125	0.25	0.5	1	2	4	8	16	32	64	128	256	512	1024	MIC$_{50}$	MIC$_{90}$
																						µg/mL	
**Tilozin**															1	0	44	0	4	13	8	64	1024
															1.4%	0.0%	62.9%	0.0%	5.7%	18.6%	11.4%		
**Tiamulin**																2	47	5	13	2	1	64	256
																2.9%	67.1%	7.1%	18.6%	2.9%	1.4%		
**Lincomycin**																	44	0	0	4	22	64	1024
																	62.9%	0.0%	0.0%	5.7%	31.4%		
**Vancomycin**																44	0	4	13	8	1	32	512
																62.9%	0.0%	5.7%	18.6%	11.4%	1.4%		
